# Middle school teachers’ implementation and perceptions of automated writing evaluation

**DOI:** 10.1016/j.caeo.2024.100231

**Published:** 2024-12

**Authors:** Joshua Wilson, Amanda Delgado, Corey Palermo, Tania M. Cruz Cordero, Matthew C. Myers, Halley Eacker, Andrew Potter, Jessica Coles, Saimou Zhang

**Affiliations:** a University of Delaware, USA; b Measurement Incorporated, USA; c Arizona State University, USA

**Keywords:** Automated writing evaluation (AWE), Automated feedback, Writing, Perceptions, Implementation

## Abstract

Despite research supporting the efficacy of Automated Writing Evaluation (AWE) in improving writing outcomes, inconsistent implementation by teachers raises concerns about the efficacy of these systems in practice. However, little is known about what factors influence teachers’ implementation and perceptions of AWE. This study examined the relationship between teachers’ implementation and perceptions of the MI Write AWE system, seeking to identify actionable factors that could enhance AWE implementation and acceptance in the future. A mixed-methods design was utilized, combining quantitative analysis of usage logs and survey data with qualitative insights from focus groups and interviews with 19 teachers who participated in a randomized controlled trial (RCT) testing the efficacy of MI Write on students’ writing outcomes. Quantitative data were subjected to descriptive and non-parametric statistical analyses, while qualitative data underwent a deductive coding process, offering an integrated view of MI Write's use and educators’ perceptions. Teachers implemented MI Write variably and not to the extent expected of them within the RCT, but they did report generally positive attitudes towards MI Write. Findings indicated that positive perceptions of system usability and usefulness may be insufficient to promote effective implementation. Instead, ecological factors such as curricular alignment and the challenge of incorporating AWE into existing workload, administrative support, and broader social and educational policy appeared as factors influencing implementation. Findings emphasize that teachers’ implementation and perceptions of AWE are dependent on a range of contextual elements beyond mere system functionality, suggesting that successful adoption requires addressing broader ecological considerations.

## 1 Introduction

Writing plays an essential role in academic success; however, most students in the United States do not meet grade-level expectations for writing proficiency [1] as defined by educational standards like the Common Core State Standards. To address this situation, researchers and educators are increasingly interested in technology-based writing interventions due to their scalability and success in improving students’ writing outcomes [2,3].

One technology-based writing intervention is automated writing evaluation (AWE), which has been the focus of increased research and attention in recent years [4,5,6]. AWE are software systems that utilize natural language processing (NLP) and artificial intelligence (AI) to evaluate students’ writing and provide students with immediate qualitative feedback [7,8,9]. AWE frequently also provides automated writing quality ratings intended to help students calibrate and measure growth relative to performance expectations. Although there remain concerns regarding the construct and consequential validity of AWE systems [10] and the transparency of the algorithms underlying AWE feedback and scoring (see [11,12]), research indicates that AWE supports improvements in students’ overall writing quality and thus shows promise as an effective technology-based writing intervention [4,13,14,15].

While some technology-based writing interventions, like intelligent tutoring systems (e.g., [16,17]), can operate independently of direct teacher involvement, many AWE systems, such as *MI Write, Criterion*, and *MY Access!*, necessitate significant teacher participation in their implementation. For those AWE systems, teachers are responsible for assigning either pre-packaged or customized writing prompts before students can engage in writing practice and receive feedback. Additionally, teachers monitor students’ activity within the system, review reports, and offer supplementary feedback to complement the automated feedback. Consequently, the effectiveness of those AWE systems in enhancing students’ writing outcomes is partly contingent upon the degree to which teachers implement them in their classrooms. As Li [18] wrote, teachers play a critical role in “facilitating or suppressing students’ use of AWE systems” (p. 2). Thus, it is crucial to understand what factors influence teachers’ AWE implementation decisions.

The Technology Acceptance Model (TAM; [19,20]) posits that system implementation hinges, in part, on users’ perceptions of the system's usability (i.e., its ease of use) and usefulness. These perceptions are believed to influence an individual's attitude toward using the system, thereby shaping their behavioral intention to use it, and ultimately their actual usage. Therefore, based on the TAM, teachers’ perceptions of AWE's usability and usefulness are hypothesized to influence how and to what extent they implement AWE.

While the TAM provides valuable insights, it overlooks various ecological factors that may also influence teachers’ perceptions of AWE. Factors such as administrative and peer support, curricular pressures and priorities, and other environmental influences may shape both how teachers perceive and implement AWE. Therefore, a comprehensive understanding of the adoption and implementation of AWE must consider these broader contextual factors alongside individual perceptions. Accordingly, the present study utilized a rich mixed methods investigation to describe how teachers implemented and perceived an AWE system called MI Write, with the goal of identifying factors that may influence teachers’ AWE implementation and perceptions. By integrating quantitative usage data, quantitative data from an AWE-perceptions survey, and qualitative insights from focus groups and interviews, this study leveraged the affordances of a small teacher sample to provide a nuanced understanding of the interplay between individual attitudes and broader ecological factors in the successful adoption of AWE in educational settings.

### 1.1 Factors influencing teachers’ implementation and perceptions of awe

AWE is commonly implemented as a formative assessment to improve students’ writing outcomes and several recent meta-analyses attest to its promise in this regard [13,14,15]. Yet, there is a paucity of research that explores teachers’ implementation and perceptions of AWE when implemented for that purpose. A search for articles published between January 2015 and the present in the Web of Science database using the topic keywords “automated writing evaluation” OR “automated essay evaluation” OR “automated essay scoring” OR “automated feedback,” yielded 556 peer-reviewed journal articles. However, only 16 articles investigated or reported data on teachers’ implementation and perceptions of AWE within classroom settings. Of those, just 5 articles focused on middle school teachers, who are the subject of the present study.

Indeed, Barrot's [4] bibliometric review of AWE research published between 2002 and 2021 found that nearly two-thirds of the 170 included documents were conducted within higher-education contexts, and less than 8 % were conducted within secondary contexts. Barrot [4] also highlighted a clear gap in the literature regarding the classroom implementation context of AWE. Without comprehensive insights into the practical application and variability of AWE usage, educators and policymakers may struggle to fully understand its potential and limitations.

Relatedly, a prior literature review by Celik et al. [21] reported nine common challenges that teachers faced when implementing AI-based educational technologies. In support of the TAM, many challenges focused on system limitations, such as reliability, technical capacity, inefficiency, or limitations in AI adaptive feedback. Another challenge related to teachers’ lack of knowledge or interest in using the AI-based systems. Only two challenges addressed broader ecological factors: lack of technical infrastructure in schools and the inapplicability of the AI system to multiple settings.

Considered in terms of AWE, Celik et al.’s [21] summary is revealing, emphasizing the importance of acknowledging system-specific limitations, particularly the limitations of AWE feedback. However, research suggests that other ecological factors also influence teachers’ AWE implementation and perceptions. These factors include administrative and peer support, curricular and instructional integration, and differential advantages by instructional task. The following sections will discuss these factors in detail, highlighting their influence on teachers’ implementation and perceptions of AWE.

#### 1.1.1 Limitations of awe feedback

Teachers consistently report that AWE feedback assists but cannot replace human feedback due to its limited ability to provide effective higher-level feedback [22,23,24,25,18,26]. For example, Correnti et al.’s [23] study of 16 fifth and sixth grade teachers in the US implementing the *eRevise* system found that while eRevise feedback showed promise, more than two-thirds of teachers indicated that at least some of their students had difficulty understanding and applying the automated feedback. Similarly, Authors [27] analyzed data from focus groups conducted with 90 teachers in Grades 3–5 and reported that teachers believed MI Write was not sensitive to the styles and voices of students’ writing; instead, they suggested individualizing the automated feedback so that it would consider students’ writing growth dynamically.

Teachers in L2 instructional contexts, particularly in higher education, also recognize both the assistive and limiting roles of AWE systems [24,25]. In general, these teachers have trusted AWE's functions for correcting mechanics and grammar errors as well as promoting some metalinguistic skills. However, they have reported the feedback offered by AWE systems like Criterion, Grammarly, and *Pigai* is sometimes inaccurate and less precise than teacher feedback [24,28,25]. L2 teachers have also criticized AWE feedback related to writing organization as formulaic, expressing concerns that this potentially hampers students’ creativity [25].

These limitations of AWE feedback highlight the need for a hybrid approach where AWE is supplemented with human feedback to provide comprehensive support to students. Some teachers may perceive such a hybrid approach as affording the opportunity to provide more higher-level feedback [29,28,26] and to afford pedagogically useful interactions with students [30]. For instance, teachers have reported that AWE helped them achieve large educational goals, including increasing the amount and immediacy of feedback, reinforcing the notion that writing is a process and revising is important, transforming teachers’ understanding of students’ needs, and bridging communication with parents [29,27,31,23]. However, other teachers find it laborious to have to correct AWE's inaccuracies and to reorient students’ perceptions of their work [27,25].

#### 1.1.2 Administrative and peer support

Administrative and peer support in AWE implementation is an important ecological factor not included in the TAM. As noted by Grimes and Warschauer [32] in their seminal study of 16 middle school teachers in three school districts, strong leadership and peer support systems for implementing AWE foster positive attitudes and greater benefits for students. For instance, school districts with robust administrative backing and organized peer support demonstrated enhanced student outcomes, increased peer collaboration, and higher assignment completion rates with the AWE tool MY Access!. Similarly, Mayfield and Butler [33]’s study of *Revision Assistant* implementation in 33 schools in 16 school districts demonstrated that greater and more successful implementation was achieved in districts where superintendents, principals, and other administrators collaborated with instructional coaches and specialists to actively support the implementation of Revision Assistant. However, in districts where leadership failed to prioritize AWE tools, teachers may struggle to integrate these systems effectively. In such cases, teachers' AWE implementation may be idiosyncratic, guided by their perceptions of the system, their beliefs about feedback and instruction, and their technological pedagogical background knowledge [28,25,18].

#### 1.1.3 Curricular and instructional integration

Another factor influencing AWE implementation is the alignment with curriculum and instruction. Warschauer and Grimes's [34] seminal study of AWE implementation collected interview data from eight teachers and survey data from seven teachers. They found that curricula prioritizing reading over composition hindered AWE adoption. Similarly, Mayfield and Butler [33] reported that effective integration involved aligning AWE with district-specific writing prompts and instructional practices. In their study, superintendents, principals, and other administrators collaborated with instructional coaches to ensure writing prompts aligned with the local curriculum, resulting in coherent integration. Palermo and Wilson [30] also identified curricular integration as a crucial factor influencing AWE implementation. Interviews conducted with 14 middle school teachers revealed that teachers attributed their successful implementation of the *NC Write* AWE system to it being well aligned to their pedagogical practices.

Teachers must navigate these curricular demands to optimize AWE benefits, tailoring their use of AWE tools to fit within the broader educational framework and specific instructional contexts. Effective AWE integration requires not only aligning with curricular priorities but also adapting to the instructional strategies employed within the classroom.

#### 1.1.4 Differential advantages by instructional task

Another factor influencing teachers’ perceptions is the differential effectiveness of AWE depending on the instructional task. For example, Grimes and Warschauer [32] showed that middle school teachers perceived conventional approaches as more suitable for teaching higher-level traits of writing (i.e., ideas, organization, voice, and word choice), whereas they perceived AWE as more suitable for teaching lower-level traits of writing (i.e., sentence fluency and conventions).

However, depending on the task, some teachers have even questioned the appropriateness of using AWE for lower-level feedback. For instance, interviews with four ESL writing instructors revealed that even though instructors generally viewed Criterion in a positive manner, they raised concerns as to whether its feedback may undermine the goal of students independently recognizing and correcting their grammar mistakes [28,25]. This variability in perceived effectiveness underscores the importance of matching AWE tools with specific instructional objectives to maximize their utility in the classroom.

As further, Wilson et al. [35] demonstrated that the variable implementation of MI Write across 24 teachers’ classrooms in Grades 3–5 appeared contingent on the assignment categories within the curriculum (e.g., genres), adaptive instructional approaches (e.g., whole-group versus independent practice), and the interaction of these factors. Results showed that teachers in Grades 3, 4, and 5 prioritized assigning persuasive, narrative, and informative writing assignments, respectively. Furthermore, fifth grade teachers utilized more prompts for whole-group instruction (50 % of assigned prompts) than third or fourth grade teachers (40 % and 28 % of assigned prompts, respectively). This suggests that instructional-level decisions, such as the preference for using AWE in whole-group versus independent practice, also significantly influence AWE implementation.

#### 1.1.5 Summary of factors influencing awe implementation and perceptions

In summary, the interplay of factors including the limitations of AWE feedback, administrative and peer support, curricular and instructional integration, and differential advantages by instructional task appear to influence teachers’ perceptions and implementation of AWE in addition to teachers’ perceptions of system usability and usefulness, as posited by the TAM.

AWE feedback often requires supplemental teacher feedback to address higher-level writing skills, clarify student misunderstandings, and correct inaccurate feedback; teachers may find that either beneficial or burdensome. Strong administrative support and peer collaboration are crucial for successful AWE implementation. Effective integration involves aligning AWE with curricular priorities and instructional practices. The differential effectiveness of AWE across writing tasks necessitates careful integration within teacher instruction, which may vary based on course goals and pedagogical beliefs. When these factors are lacking, AWE implementation may suffer, and teachers may hold negative perceptions of AWE.

Despite these insights, research on teachers’ implementation and perceptions of AWE is limited. Thus, this summary is not exhaustive. The complexity of classroom environments and the diversity of instructional contexts suggest that additional, yet unidentified factors may influence AWE success.

### 1.2 Study purpose

Despite increasing interest in AWE as a promising technology-based writing intervention [4,5,6], and despite recent advancements in the NLP and AI architecture undergirding AWE systems (see [36,37]), there remains a significant gap in research specifically focused on how teachers implement and perceive AWE in classroom settings. The lack of detailed research in this area poses challenges for researchers, educators, and policymakers in fully understanding and leveraging AWE's potential benefits. If AWE systems are difficult to implement, inconsistently used, or not fully embraced by teachers, their potential to improve student writing outcomes cannot be fully realized.

This study addresses this research gap by examining the relationship between teachers’ implementation and perceptions of the MI Write AWE system, a system that requires considerable teacher involvement. Utilizing a rich mixed-methods research design that leverages the affordances of a small sample size, this study examines usage, survey, and qualitative data from 19 middle school English language arts (ELA) teachers who participated in a randomized controlled trial (RCT) that tested the effectiveness of writing instruction when integrated with MI Write versus business-as-usual writing instruction where students lacked access to MI Write.

This study investigates how the extent of teachers’ MI Write implementation relates to their perceptions about its usability, usefulness, and desirability as an instructional tool. Study findings are intended to provide valuable insights for stakeholders, identifying factors that may influence successful implementation and suggesting ways to align AWE tools more closely with teachers’ needs and perceptions.

Two research questions guided this study:

RQ1 To what extent do teachers implement MI Write's features and overall? Were there differences across school districts?

Our first question leverages quantitative usage data collected in MI Write log files. We anticipated variability in usage across teachers and districts (e.g., [35,33]).

RQ2 To what extent and in what ways do teachers perceive MI Write as a usable, useful, and desirable tool for the teaching of writing? Do teachers’ perceptions of MI Write differ across districts?

This research question deliberately combines quantitative (i.e., to what extent) and qualitative (i.e., in what ways) components [38,39]. We examined these perceptions given their theoretical importance within the TAM [19,20] and given their salience in prior research (e.g., [31,40]). Based on prior research, we hypothesized that teachers would report generally positive perceptions but anticipated variability across teachers and potentially across districts.

## 2 Methods

### 2.1 Study context

Data for the present study were collected with IRB approval as part of a larger Bill and Melinda Gates Foundation funded randomized controlled trial (RCT) studying the impact of MI Write on teacher and student outcomes. The Gates Foundation was interested in testing the efficacy of well-established AWE systems, like MI Write (see Section 2.2), as part of an initiative aimed at improving writing outcomes for middle school students from historically disadvantaged demographic populations in the U.S.

The RCT was conducted in the 2021–2022 school year and involved three school districts in the Mid-Atlantic and Southern regions of the United States who had not previously implemented any AWE system. District A, located in a suburban setting, enrolls approximately 10,500 students and has two middle schools, where 48 % of middle school students pass the state ELA assessment. District B, located in an urban setting, enrolls nearly 27,000 students, has 21 middle schools, and 50 % of middle school students pass the state ELA assessment. District C, located in a rural setting, enrolls around 10,000 students, and has five middle schools, where 26 % of middle school students pass the state ELA assessment. District A followed a district-specific writing curriculum that focused on teaching one purpose/genre per marking period, starting with narrative and concluding with literary analysis. On the other hand, both Districts B and C adopted the StudySync curriculum, a web-based ELA curriculum with six units covering various writing purposes/genres, such as argumentative, informative, explanatory, literacy analysis, and narrative.

Within these districts, all Grade 7 and 8 English Language Arts (ELA) teachers were invited to participate in the study. Teachers were only excluded if they did not teach any “general” ELA sections, that is, if they only taught students with disabilities or only taught students who were English language learners. Among those who consented to be in the study, teachers were randomly assigned to either the treatment group (*n* = 19) or a comparison group (*n* = 18). Teachers in the treatment group utilized the MI Write AWE system to support their ELA instruction throughout the year, following implementation expectations provided by the research team—see Section 2.2.1. Teachers in the comparison group delivered their standard ELA instruction without the use of MI Write, resulting in a “business as usual” comparison to the MI Write treatment group. Collectively, these teachers instructed more than 2500 seventh and eighth grade students who also participated in this RCT, making this one of the largest impact studies of AWE to date.

Randomization was performed at the teacher level through random number generation and accounted for nested data by blocking teachers by district, school, and grade. Data collection for this study took place in late May/early June, involving electronic surveys collected via Qualtrics. Post-academic year, treatment teachers had the option to participate in focus groups or interviews to discuss their experiences and perceptions of MI Write.

During the study, the COVID-19 pandemic continued to affect schools and student learning. Educators faced additional challenges such as a mix of in-person and remote instruction, extensive teacher and student absences, the need to make-up missed district and state testing from Spring 2021, teacher burnout, and concerns about students’ learning loss.

### 2.2 MI Write

Developed and marketed by Measurement Incorporated, MI Write (https://miwrite.com/#contact), previously called *PEG Writing*, is an AWE that facilitates writing instruction and learning in the classroom by providing immediate, automated feedback and scores in response to student writing [31]. MI Write can be used with both pre-packaged, system-created writing prompts as well as teacher-created, customized writing prompts.

MI Write leverages the *PEG* (Project Essay Grade) automated scoring engine to evaluate six traits of writing quality (i.e., the Six Trait Model; [41]): idea development, organization, style, sentence fluency, word choice, and conventions (range for each trait = 1.0–5.0). MI Write also produces a holistic score (range = 6.0–30.0), which is formed as the sum of the six trait scores. To produce these automated quality ratings, PEG conducts feature extraction of more than 800 linguistic variables that are both theoretically and empirically associated with writing quality. Scoring models for six writing traits across three genres (narrative, argumentative, and informative/explanatory) and five grade bands (3–4, 5–6, 7–8, 9–10, 11–12) are built to assess writing quality without being constrained to evaluating specific prompts. Thus, training samples include varied prompts within each genre and grade band. A stepwise training approach optimizes each model, resulting in scores consistent with human ratings (average quadratic weighted kappa in the low .800′s for each trait*genre*grade-band model).

Although PEG utilizes nearly 800 linguistic features to build its scoring models, MI Write is designed to prioritize both scoring accuracy and interpretability. Therefore, a limited set of instructionally relevant features are selected from which MI Write's automated feedback is generated. PEG identifies feedback features likely to yield the greatest gains in writing quality. The model's architecture ensures that score improvements occur when students revise an essay to enhance positive features or reduce negative ones. Automated feedback from MI Write takes the form of metacognitive prompts to help students evaluate their writing (e.g., *Does your writing have a clear conclusion?*) as well as suggestions for improvement (e.g., *Many of your sentences are very simple. Try to combine sentences or use more introductions to add more information and create more interesting sentences*). MI Write additionally provides students with in-line feedback regarding spelling and grammar errors.

MI Write also has a suite of interactive, multimedia skill building lessons that are suggested to students based on their writing performance. Lessons address writing skills related to each of the six traits (39 lessons total across traits), grammar (41 lessons), and editing a passage of writing (25 lessons). Lessons are short, ranging generally from 2 to 6 min, but occasionally longer 10–14 min, and involve narrated animations and interactive activities.

MI Write also includes peer review functionality, which may be conducted as either anonymous, single blind (i.e., either the author or the reviewer is identified), or fully identifiable peer review (i.e., both the author and reviewer are identified). To scaffold peer review, teachers customize a peer review rubric within MI Write by selecting among a menu of critical questions (e.g., “Does the writer thoroughly support the claim with valid reasons and relevant evidence from credible sources?”) that are aligned to grade-level genre-specific writing expectations. Students then seek to answer those questions when evaluating their peer's writing, assigning both a numeric score ranging from 0 (not present) to 2 (fully present) and an associated qualitative comment explaining their evaluation, offering suggestions when appropriate.

Finally, in MI Write, teachers can correspond with students via messages and supplement MI Write's feedback with in-line and summary comments in response to student writing. MI Write also provides students and teachers with several reporting features that allow for tracking students’ activity and progress over time, identifying individual and classwide areas of improvement, differentiating instruction, and setting goals and monitoring progress towards those goals.

The Gates Foundation selected MI Write for an efficacy trial because of prior research indicating MI Write's promise for improving students’ writing quality [42,30], students’ writing motivation and self-efficacy for writing [29,43], and its widespread adoption in the U.S. (e.g., [42,27,31]).

#### 2.2.1 Implementation expectations (Fidelity)

After receiving training, teachers in the treatment group (*n* = 19) were expected to implement MI Write throughout the school year to support their ELA instruction. To guide their implementation, the research team provided treatment teachers with a set of researcher-created fidelity expectations informed by prior empirical studies. Fidelity expectations stipulated that treatment teachers should engage with MI Write monthly from October to May, encompassing a range of activities. Specifically, teachers were to assign eight writing prompts within MI Write, each with one of MI Write's electronic graphic organizers; assign eight MI Write multimedia lessons; assign peer-review within MI Write for three writing assignments from March to May; and provide supplemental teacher feedback to complement the automated feedback on at least five student assignments from January to May. Meeting these criteria was considered adherence to fidelity expectations. To facilitate this, MI Write coaches offered both scheduled and on-demand training during the implementation phase.

These fidelity expectations were developed based on prior research indicating that positive outcomes occurred when six essays were completed within MI Write [42,30], as opposed to non-effects observed when only three essays were completed with MI Write [29]. In addition, the fidelity expectations emphasized distributed practice across the academic year, aligning with research indicating its superiority for skill maintenance [44]. That is, the fidelity expectations emphasized consistent monthly practice (i.e., 1 writing assignment per month) rather than massing all the writing practice towards the end of the year. Finally, the fidelity expectations included all major MI Write features—assignments, graphic organizers, multimedia lessons, peer review, teacher feedback—based on evidence that teachers underutilize specific features in naturalistic settings [45,31]. Finally, these fidelity expectations were balanced against the need to minimize teacher burden.

### 2.3 Participants

#### 2.3.1 Quantitative sample

The quantitative sample consisted of the 19 teachers randomly assigned to the MI Write treatment condition: 6 teachers were from District A, 10 were from District B, and 3 were from District C. All 19 treatment teachers completed a survey regarding their perceptions of MI Write's usability, usefulness, and desirability as a pedagogical tool. Table 1 presents teacher demographics overall and disaggregated by district. There were no differences in demographic characteristics across districts except for the percentage of teachers possessing a Masters degree or higher and average years teaching (District *B* > *A* = C). None of the teachers used MI Write or a different AWE system before.

**Table 1:** Teacher Demographics.

	Full sample (*n* = 19)	District A (*n* = 6)	District B (*n* = 10)	District C (*n* = 3)	
Gender					*X^2^*_(2)_ =0 0.35, *p* = 0.840
Male	21.05 % (4)	16.67 % (1)	20 % (2)	33.33 % (1)	
Female	78.95 % (15)	83.33 % (5)	80 % (8)	66.67 % (2)	
Race					*X^2^*_(2)_ = 14.64, *p* = 0.067
White	36.84 % (7)	50 % (3)	20 % (2)	66.67 % (2)	
Black	21.05 % (4)	0 % (0)	30 % (3)	33.33 % (1)	
Hispanic	5.26 % (1)	16.67 % (1)	0 % (0)	0 % (0)	
Multiracial	10.53 % (2)	33.33 % (2)	0 % (0)	0 % (0)	
Prefer not to say	0 % (0)	0 % (0)	50 % (5)	0 % (0	
Masters degree or higher	42.11 % (8)	16.67 % (1)	70 % (7)	0 % (0)	*X^2^*_(2)_ = 6.97, *p* = 0.031
Years teaching (*M, SD*)	12.95 (7.31)	8.33 (6.28)	17.90 (4.61)	5.67 (4.51)	*F*_(2,16)_ = 9.92, *p* = 0.002

*Note.* Frequencies presented in parentheses.

#### 2.3.2 Qualitative sample

Due to the high level of participatory workload required from teachers during the RCT and to reduce the risk of participant burnout during a school year still affected by the COVID pandemic, all teachers were invited, but not required, to participate in the qualitative data collection and were offered a financial incentive to do so. Nine teachers chose to participate in either an individual interview (*n* = 5) or a focus group (*n* = 4), with representation from all three school districts. Interviews and focus groups were conducted and audio-recorded by trained graduate research assistants using standardized administration protocols.

### 2.4 Measures

#### 2.4.1 MI Write usage indicators

Teacher usage data were extracted from MI Write log files, which measured all activity in MI Write during the school year. Usage indicators derived from log files included the number of graphic organizers, prompts, lessons, and peer reviews teachers assigned, as well as the number of student essays teachers annotated.

#### 2.4.2 AWE perceptions scale (AWE-P)

The *AWE-P* [35,46] is a researcher-developed 19-item electronic survey comprised of items scored on a four-point Likert scale ranging from 0 (Strongly Disagree) to 3 (Strongly Agree). The scale contained three subscales: Usability, Usefulness, and Social Desirability. All teachers completed the AWE-P, resulting in 0 % missing data. The AWE-P was validated in the 2020–21 academic year, prior to deployment in the current RCT, after piloting the survey with 60 teachers from a school district in the Southwestern U.S. and confirming that its three subscales were sufficiently reliable (Wilson, unpublished data).

##### 2.4.2.1 Usability scale

The Usability Scale was comprised of seven items that assessed teachers’ perceptions on how easy it was to use MI Write as a whole (e.g., *Overall, it is easy to use MI Write*) as well as its different features (e.g., *Assign lessons to my students; Create my own prompts*). To avoid missing data, each item was accompanied by a response that stated, “I did not use this feature.” The Usability Scale was highly reliable (Cronbach's alpha [α] = 0.92).

##### 2.4.2.2 Usefulness scale

The Usefulness Scale was comprised of ten items that gauged teachers’ perceptions of MI Write's effects on writing instruction and students’ learning. Items assessed whether teachers thought MI Write supplemented and assisted in the teaching process (e.g., *MI Write alleviated my grading load; MI Write is useful for class wide progress monitoring*) and whether students’ writing skills improved because of using MI Write (e.g., *MI Write helps students become better writers; MI Write increases my students’ motivation to write*). The Usefulness Scale was highly reliable (α = 0.95).

##### 2.4.2.3 Social desirability scale

The Social Desirability Scale consisted of two items that assessed the extent to which teachers would recommend and use MI Write (i.e., *I would recommend MI Write to other teachers; I would like to continue using MI Write*). The Social Desirability Scale had high reliability (α = 0.86).

#### 2.4.3 Interviews and focus groups

Participants self-selected into either individual focus groups or interviews. Both formats used the same semi-structured interview protocol that aligned with the constructs measured quantitatively in the survey. The protocol was adapted specifically for the present study from one used in prior research [27]. Teachers were asked open-ended questions about MI Write's usability (e.g., *Do you think MI Write is easy to use? Why or why not?*), and usefulness (e.g., *In what ways, if any, was MI Write useful for you as a teacher?*). Teachers were also asked open-ended questions about the facilitators and barriers for implementing MI Write in their classrooms (e.g., *What were the barriers impacting your ability to implement MI Write in your classroom?*) and how they implemented and used MI Write in the classroom (e.g., *How did you have your students approach revising through MI Write?*). A copy of the protocol is provided in the Appendix. Interviews and focus groups were conducted and audio-recorded via Zoom by trained graduate research assistants using standardized administration protocols. Transcripts generated from Zoom were cleaned and then used for qualitative analysis.

### 2.5 Data analysis

Descriptive statistics summarized teachers’ MI Write implementation as well as teachers’ perceptions of MI Write based on the AWE-P Usability, Usefulness, and Social Desirability subscales. Due to the small sample size (*n* = 19), ANOVA and follow-up post hoc comparisons were deemed inappropriate for examining whether there were statistically significant differences in teachers’ implementation and perceptions across districts. Instead, we adopted the non-parametric alternative, specifically the Kruskal-Wallis test and follow-up comparisons using Dunn's test. This decision to use non-parametric analyses also better aligned with the descriptive nature of our analyses, as we aimed to describe differences among our sample rather than test hypotheses about a larger population.

We analyzed qualitative data from individual interviews and focus groups using a deductive coding approach to further explore teachers’ ratings. We assigned predetermined codes related to MI Write's usability, usefulness, and desirability to interview transcriptions. To ensure the reliability of our coding process, we employed consensus coding, where multiple researchers independently coded the data and then convened to discuss and reconcile any discrepancies, thus achieving inter-coder reliability. From the consolidated codes, we systematically identified patterns and relationships, which facilitated the generation of themes that encapsulate the overarching insights related to teachers’ perceptions of MI Write.

### 2.6 Mixed methods study design

The current study employed a convergent parallel design with concurrent quantitative and qualitative strands of data collection [47]. A convergent parallel design achieves “a more complete understanding of a problem, to validate one set of findings with the other, or to determine if participants respond in a similar way if they check quantitative predetermined scales and if they are asked open-ended qualitative questions” [47].

Additionally, this mixed methods study focused on triangulation to evaluate the degree of convergence of different sources of data that measure the same set of constructs—teachers’ perceptions of AWE's usability, usefulness, and desirability. We collected qualitative data through semi-structured interviews and focus groups to expand on the results of the quantitative data collected from surveys, as relying solely on survey data would have limited the ability for respondents to fully express their perspectives [48]. Additionally, combining quantitative and qualitative data offer a more nuanced understanding of teachers’ perceptions and helps identify convergences between results, which are more credible than results obtained using a single method [49].

#### 2.6.1 Integration

The current study incorporates both quantitative and qualitative data for the purpose of providing a comprehensive understanding of the data [47]. Qualitative data was the dominant aspect in our research design (i.e., quan + QUAL). Integration of the methods occurred in two ways as outlined by Fetters [50]. First, methods were integrated through *connection* as participants from the interview portion were selected from a sample of participants that completed the surveys. Second, integration occurred through *merging*, which took place after quantitative and qualitative data collection and consisted of bringing the two types of data together for analyses and comparison. In the current study, the questions asked in the interview and focus groups were aligned with the constructs and items addressed in the AWE-P survey. Data were also integrated through a *narrative* approach that described the quantitative and qualitative results thematically. Specifically, we used a *weaving approach*, where both quantitative and qualitative findings were written together on a theme-by-theme basis [50].

## 3 Results

### 3.1 RQ1: teachers’ implementation

Table 2 displays the extent to which teachers on average used and assigned MI Write activities during the school year overall and by district. There were no significant differences across districts regarding total MI Write usage, as indicated by the Kruskal-Wallis test [H_(2)_ = 4.143, *p* = 0.126], and none of the teachers in the study met the fidelity target. However, teachers implemented certain MI Write components more consistently than others. For instance, although fewer than half (42.1 %) of teachers assigned the expected eight graphic organizers and prompts, teachers assigned an average of 7.26 separate organizers and prompts (range = 2–16), just shy of the fidelity target. There were no significant differences among districts in the average usage of organizers [H_(2)_ = 2.035, *p* = 0.361] and prompts assigned [H_(2)_ = 2.035, *p* = 0.126].

**Table 2:** Fidelity Expectations and Descriptive Statistics of Teachers’ MI Write Implementation Overall and by District.

	Fidelity expectation	Overall sample		District A	District B	District C	Kruskal-Wallis H (*df* = 2)	Dunn's post hoc test
Usage variables		*M* (*SD*)	Mode	*M* (*SD*)	*M* (*SD*)	*M* (*SD*)		
Organizers assigned	8	7.26 (3.41)	7, 9	5.67 (3.20)	8.00 (3.80)	8.00 (1.73)	2.035	*A* = *B* = C
Prompts assigned	8	7.26 (3.41)	7, 9	5.67 (3.20)	8.00 (3.80)	8.00 (1.73)	2.035	*A* = *B* = C
Lessons assigned	8	4.89 (4.33)	3	3.00 (1.41)	4.30 (4.40)	10.67 (3.79)	5.798	*A* = *B* = C
Prompts with peer review assigned	3	1.58 (3.08)	0	2.33 (5.24)	1.00 (1.63)	2.00 (1.00)	2.919	*A* = *B* = C
Prompts with annotations	5	0.65 (1.03)	0	0.50 (0.84)	0.20 (0.42)	2.00 (1.73)	6.600*	*A* = *B* < C = A

Total MI Write tasks assigned	32	21.58 (9.38)	14, 18, 26, 29	17.17 (10.80)	21.50 (8.29)	20.67 (2.89)	4.143	*A* = *B* = C

*Note*. A prompt refers to a unique writing assignment created by the teacher. An essay is an individual student's response to that prompt. Dunn's post hoc test conducted using a Bonferroni correction to alpha. *p ≤ 0.05.

On average, teachers assigned 4.89 lessons (range = 0–16), and only 21.1 % of teachers met fidelity expectations in assigning eight interactive lessons in MI Write. Kruskal-Wallis test results indicated no significant differences across districts in lesson assignment [H_(2)_ = 5.798, *p* = 0.055].

Teachers least often utilized the peer review and annotation functions of MI Write, which is consistent with prior research of MI Write [45,31]. Teachers assigned an average of 1.58 peer review assignments throughout the year (range = 0–13). Similarly, on average, teachers annotated only 0.58 prompts (range = 0–4). This indicates that the average teacher did not assign any peer review activities within MI Write or use MI Write's annotation feature to provide written feedback to students. Fidelity expectations for these functions were lower (i.e., 3 and 5, respectively) because teachers were not trained to use these functions at the start of the study, but over time. Only 15.79 % of teachers met fidelity expectations for assigning peer review and 0 % met fidelity expectations for using the annotation tool. As shown in Table 2, there were no significant differences across districts in the average number of prompts assigned with peer review [H_(2)_ = 2.919, *p* = 0.232], but there was a significant difference in the use of the annotation tool [H_(2)_ = 6.600, *p* = 0.0369]. Results of post hoc tests using Dunn's multiple comparison test with a Bonferroni correction indicated that teachers in District C made greater use of the annotation tool than teachers in District B; there were no differences in use between Districts A and B, nor between Districts A and C.

### 3.2 RQ2: teachers’ perceptions

Table 3 presents the descriptive statistics of the AWE-P for teachers’ perceptions of MI Write usability, usefulness, and desirability across districts, as well as by district.

**Table 3:** Teacher Perceptions of MI Write's Usability, Usefulness, and Social Desirability.

		Overall		District A	District B	District C
	*M* (*SD*)	Median	Mode	*M* (*SD*)	*M* (*SD*)	*M* (*SD*)
Usability – *It is easy to…* (Scale)	2.16 (0.55)	2.00	2.00	1.68 (0.34)	2.35 (0.41)	2.44 (0.82)
1. Create my own prompts	2.44 (0.51)	2.00	2	2.17 (0.41)	2.56 (0.53)	2.67 (0.58)
2. Assign a writing prompt	2.42 (0.61)	2.00	2, 3	2.00 (0.63)	2.60 (0.52)	2.67 (0.58)
3. Use annotation tool	2.06 (0.75)	2.00	2	1.67 (0.52)	2.38 (0.74)	2.00 (1.00)
4. Assign lessons	2.22 (0.65)	2.00	2	1.67 (0.52)	2.44 (0.53)	2.67 (0.58)
5. Set up peer review	2.00 (0.73)	2.00	2	1.60 (0.55)	2.13 (0.64)	2.33 (1.16)
6. Interpret score reports	1.82 (0.95)	2.00	2	1.00 (0.89)	2.25 (0.46)	2.33 (1.16)
7. Use MI Write overall	1.89 (0.81)	2.00	1, 2	1.17 (0.41)	2.20 (0.63)	2.33 (1.16)
Usefulness – *MI Write…* (Scale)	1.83 (0.63)	1.84	1.00, 3.00	1.17 (0.21)	2.10 (0.37)	2.33 (0.88)
1. Helps provide more writing practice	2.16 (0.69)	2.00	2	1.50 (0.55)	2.40 (0.52)	2.67 (0.58)
2. Alleviates my grading load	1.47 (0.91)	1.00	1	0.67 (0.52)	1.70 (0.82)	2.33 (0.58)
3. Lets me focus on feedback on higher-level writing	1.84 (0.69)	2.00	2	1.17 (0.41)	2.20 (0.42)	2.00 (1.00)
4. Useful for classwide progress monitoring	1.89 (0.66)	2.00	2	1.33 (0.52)	2.10 (0.32)	2.33 (1.16)
5. Feedback addresses student individual needs	1.84 (0.77)	2.00	2	1.17 (0.75)	2.10 (0.32)	2.33 (1.16)
6. Helps me tailor instruction to students’ writing needs	1.84 (0.77)	2.00	2	1.17 (0.41)	2.10 (0.57)	2.33 (1.16)
7. Useful for monitoring progress of individual students	1.95 (0.62)	2.00	2	1.50 (0.55)	2.10 (0.32)	2.33 (1.16)
8. Increases students’ motivation to write	1.26 (0.93)	1.00	1	0.50 (0.55)	1.60 (0.70)	1.67 (1.53)
9. Helps students learn characteristics of good writing	2.00 (0.58)	2.00	2	1.67 (0.52)	2.00 (0.47)	2.67 (0.58)
10. Helps students become better writers	2.05 (0.62)	2.00	2	1.50 (0.55)	2.20 (0.42)	2.67 (0.58)
Social Desirability	1.87 (0.91)	2.00	1.00, 3.00	0.83 (0.26)	2.30 (0.63)	2.50 (0.87)
1. I would recommend MI Write to other teachers	1.95 (0.91)	2.00	2	0.83 (0.41)	2.40 (0.52)	2.67 (0.58)
2. I would like to continue using MI Write	1.79 (1.03)	2.00	1, 3	0.83 (0.41)	2.20 (0.92)	2.33 (1.16)

*Note*. Responses to all items were recorded on a 0 (Strongly Disagree) to 3 (Strongly Agree) Likert scale.

#### 3.2.1 Usability

As shown in Table 3, overall, teachers agreed that MI Write was easy to use (*M* = 2.16, *SD* = 0.55, Median = 2.00). However, there were significant differences in usability scores across districts, as indicated by the Kruskal-Wallis test [H_(2)_ = 6.535, *p* = 0.038]. Results of post hoc analyses using Dunn's multiple comparison test with a Bonferroni correction indicated that teachers in District A expressed significantly less positive usability ratings compared to teachers in District B (*p* = 0.047), but not compared to teachers in District C (*p* = 0.192). There were no significant differences between teachers in Districts B and C (*p* = 1.000). These comparisons are based on rank sums. Additionally, the median ranks suggest that usability ratings were highest in District C, followed by District B, and lowest in District A, which is consistent with the means displayed in Table 3.

Teachers commented on MI Write's usability in positive ways. Examples included that it was “much easier and much faster” to ask students to complete essays in MI Write than on paper. Another teacher echoed this perspective, stating that they “really actually enjoyed . . . the ability to give [students] feedback so quickly, and for [students] to respond back to me.”

Specifically, teachers most strongly agreed that it was easy to create and assign writing prompts in MI Write (see Table 3). Indeed, one teacher commented:

*I feel like MI Write allowed me to have to prep less and I felt like I was prepping less because everything was already done, with the exception of coming up with the prompts…sometimes I created my own or sometimes I took the prompts from our curriculum.*

Although teachers generally agreed that MI Write was easy to use, teachers reported varied perceptions regarding specific MI Write features, in particular the peer review feature and the annotation tool. Teachers in Districts B and C, who in general expressed positive MI Write perceptions, tended to characterize the peer review feature as user friendly and useful, and were surprised by students’ engagement. One teacher from District C stated that even though students were competitive about earning the highest score, students “were still helping each other out.” Teachers continued to say they were surprised by students’ dedication to give each other feedback:

*I really thought that they were just going to click and just be like ‘whatever,’ you know. Or ‘It looks good.’ Like that's not helpful. They were really helpful, and the peer review allowed them to facilitate that better than my checklist that I used to have, or, you know, my standards of their peer review. So, they were doing it—they were helping to carry the load that I usually do and [give] specific [feedback], so I really liked that.*

Although some teachers found the peer review feature alleviated their workload and facilitated the process of assigning feedback, some teachers noted difficulties. One teacher from District B stated, “Peer review gave me a little bit of a tough time—the navigation of it, the assigning of it.” Teachers from District A appeared to face more difficulties than benefits regarding the peer review feature. One teacher said, “Students just had trouble navigating the program and accessing it individually. And when I had a whole group of 25 kids…and 15 were struggling, it was very overwhelming.” A second teacher from District C stated, “I could not figure out how to get [peer review] enabled, even though I went through all of the steps.”

Teachers across the three districts also reported difficulties interpreting and accessing the reports available within MI Write. Indeed, a teacher from District B commented that “[students] had a hard time interpreting [the reports],” and wished MI Write's score reports were “a little more student friendly.” Similarly, a teacher in District A discussed having to correct students’ misconceptions about the scores generated by MI Write. The teacher attributed this issue in part to MI Write's inability to evaluate the content of students’ writing:

*You know it's a computer, so it can only do so much. And then, you know, some kids would get a score, and it would say ‘Hey, you're at the—I don't know— 94th percentile’ and they'd be like ‘Oh my God! This is great—like, I have a 94!’ And I'd have to explain like, ‘No, no. This is a program, and it doesn't know the content—it doesn't know the task that you're working with.’ So that was a little bit of a difficulty for me because, you know, how to tell kids like ‘Sorry, that's not what the score is.’*

Teachers expressed varied perceptions about the annotation tool as well. Although some teachers indicated that the comment feature itself was easy to use, some also expressed that the feedback process did not align with their preferred method for communicating with students. For example, a teacher in District A commented:

*Most teachers like to conference in person . . . because most of our students—I have to be honest—don't read those comments. So, we're just making comments for the sake of providing the feedback via the program and the [research study's] requirement.*

Other teachers had a more positive experience. One teacher in District B said, “the kids actually looked forward to seeing what the notes were” for the purpose of revising their writing for a higher score. Such examples further highlight contextual differences among the districts.

Thus, according to the survey and interview results, teachers across districts generally perceived MI Write as being, “not very difficult to use but not super, super user friendly either,” with some features being easier to use than others and some features better aligning with a district's preferred methods of instruction.

#### 3.2.2 Usefulness

Teachers tended to agree that MI Write was useful for various aspects of writing instruction and providing students with feedback (*M* = 1.83, *SD* = 0.63, Median = 1.84). However, an equal number of teachers disagreed and strongly agreed that MI Write was useful (see Table 3). These split ratings tended to reflect the significant differences across districts with respect to usefulness, as indicated by the Kruskal-Wallis test [H_(2)_ = 11.009, *p* = 0.004]. Results of post hoc analyses using Dunn's multiple comparison test with a Bonferroni correction indicated that teachers in District A expressed significantly less positive usefulness ratings compared to teachers in District B (*p* = 0.006) and District C (*p* = 0.037). There were no significant differences between teachers in Districts B and C (*p* = 1.000). These comparisons are based on rank sums. Additionally, the median ranks suggest that usability ratings were highest in District C, followed by District B, and lowest in District A, which is consistent with the means displayed in Table 3.

Teachers most strongly agreed that MI Write was useful in providing students with more opportunities to practice writing (see Table 3). Teachers reiterated this belief in interviews, with one teacher saying:

*I would not have done as many essays this year if I was not part of the program. However, the fact that I was able to go back and have them do the same types of essays twice gave them a stronger foundation because, again, they have never seen or known how to write an essay, so they [had] more than enough practice. We literally bridged that gap.*

Teachers also reported that MI Write helped students learn characteristics of good writing and helped students become better writers. Teachers attributed this to students’ engagement during the revision process, such as the teacher who reflected that “[students] would go back and really . . . try to figure out, ‘Okay, what do I need to do to get my score up.’” Teachers also discussed how students would approach MI Write as a game in which they were competing to achieve higher writing scores:

*I think the kids are competitive by nature, so they would see how ‘I got three stars in this scale. Let me go back and work on getting four stars or three and a half stars.’ It kind of motivated them to go back, and I would see the light in their eyes, like, ‘Oh look, I have four stars this time.’ I'm like ‘Awesome!’*

Although some students were motivated to improve their writing quality scores within MI Write, this appeared not to translate to a global motivation to improve their writing skills beyond the specific tasks required by MI Write. Indeed, across districts, teachers had the least agreement that MI Write increased students’ motivation to write. One teacher suggested that students’ attitudes pointed to a larger challenge related to motivating students. This teacher stated that students consistently did not use MI Write and elaborated that the students who started off demotivated at the beginning of the year “still didn't care at the end, but that [had] nothing to do with [MI Write].” Likewise, another teacher noted that stronger writers tended to “[want] to know how to improve,” whereas weaker writers just wanted to finish the assignment.

However, there were exceptions. For instance, a teacher in District C agreed that MI Write motivated even reluctant writers, stating:

*By the end of the year, they were proud of their own accomplishments, [regardless of] how big or how small it was. MI Write was able to show them just by the data their progress. So at least they had a little bit of confidence in their abilities. I am thinking once they go to high school, maybe they can carry that with them—that they'll get back exactly what they give.*

Teachers also tended to disagree that MI Write helped alleviate their grading load, despite this being one of the intended purposes of AWE. Teachers attributed an increased workload to difficulty interpreting students’ writing scores relative to their own, district-specific criteria for writing quality. One teacher reported “doing extra work to make sure that everything [was] aligned,” and often questioned the accuracy of MI Write's scoring.

In sum, teachers tended to agree that MI Write was useful, but not for all the purposes AWE was meant to fulfill. Teachers generally agreed that MI Write provided students with more writing opportunities and that students’ writing skills improved after using the program; however, teachers generally disagreed that MI Write increased students’ motivation for writing or alleviated their grading load. Perceptions of usefulness differed across districts, with teachers in District A finding the least benefit in MI Write.

#### 3.2.3 Social desirability

As shown in Table 3, on average, teachers tended to agree that MI Write was a desirable pedagogical tool (*M* = 1.87, *SD* = 0.91, Median = 2.00), but there was a bimodal distribution with an equal number of teachers disagreeing and strongly agreeing about MI Write's social desirability. This bimodal distribution reflected the significant differences in social desirability ratings by district, as indicated by the Kruskal-Wallis test [H_(2)_ = 11.206, *p* = 0.004]. Results of post hoc analyses using Dunn's multiple comparison test with a Bonferroni correction indicated that teachers in District A expressed significantly less positive desirability ratings compared to teachers in District B (*p* = 0.007) and compared to teachers in District C (*p* = 0.027). There were no significant differences between teachers in Districts B and C (*p* = 1.000). These comparisons are based on rank sums. Additionally, the median ranks suggest that usability ratings were highest in District C, followed by District B, and lowest in District A, which is consistent with the means displayed in Table 3.

Overall, teachers generally agreed that they would recommend MI Write to other teachers and would like to continue using MI Write. Teachers’ perceptions as captured by the AWE-P survey indicated that teachers in District A were not as responsive as teachers in Districts B and C to MI Write, prompting further exploration into why there were such stark differences among districts.

Teachers from District B were more receptive to MI Write and expressed that *how* MI Write was integrated into their curriculum influenced how they accepted and implemented the program. One teacher discussed how the overlap between MI Write and the StudySync curriculum facilitated the transition to MI Write:

*Because it's so easy to transition, that makes you want to use it. If it's an addition, and not an inclusion, that makes it hard for a lot of us because then you don't, you know, it's just one more thing. But it was never one more thing—it was a part of [instruction].*

A teacher from District C echoed similar reasoning for being able to implement MI Write in the classroom. This teacher noted that the teaching materials focused on the same writing skills that MI Write supports, as exemplified by MI Write lessons:

*I didn't have to go and look for lessons or try to talk about [writing skills]—it was right there. I could just introduce [a topic] and then allow them to practice and then wrap it up and implement. So that was super easy.*

Similarly, the challenge of negotiating district-level curricular demands and the demands of MI Write implementation imposed by the RCT appeared to negatively influence teachers’ perceptions of MI Write's social desirability. For example, one teacher noted that their struggle with implementing MI Write was directly linked to the introduction of a new curriculum:

*The only problem we had this year—we probably could not have forecast this—was . . . simultaneous to having MI Write, we have a new curriculum, and that was, it turned out to be, the curriculum, turned out to be very difficult to use.*

Teachers in District A felt that the MI Write implementation expectations conflicted with the goals of their curriculum, which emphasized reading. This teacher went on to say:

*We all tried our best to hit those marks and those requirements, but I think that a lot of them were kind of unattainable because it made the focus of everything being the writing, which is totally understandable for the program itself, but not everything in our classroom is about writing.*

According to one teacher in District A, the implementation expectations for MI Write were excessive for students, as “having a writing requirement that required them to write once a month [was] just overwhelming for them.” These same requirements proved to be overwhelming for teachers, who felt burdened by the demands of “what our supervisors want us to complete and then on top of that, what MI Write wants us to complete on month-to-month basis.”

In sum, teachers’ social desirability perceptions appeared to be influenced by broader contextual factors, including alignment of MI Write with their curriculum and the resulting ease or challenge of implementing MI Write within that curriculum. In addition, social desirability was influenced by general feelings of burden arising from needing to balance learning to implement a new curriculum (StudySync) and a new technology program (MI Write), such as in District B.

## 4 Discussion

Leveraging data from an RCT testing the efficacy of the MI Write AWE system, this study sought to better understand the relationship between teachers’ implementation and perceptions of AWE and to identify factors that may influence that relationship. Identifying these factors may help researchers, AWE developers, and education stakeholders (e.g., districts considering adopting AWE) better understand the conditions necessary for realizing the benefits of AWE documented in many reviews and meta-analyses (e.g., [4,13,5,6,14]).

Before discussing the study findings, it is important to acknowledge the primary limitation of having a small sample size of 19 teacher participants, nine of whom participated in the interviews and focus groups. Although this sample size is consistent with, and even larger than, other mixed-methods research studies of AWE that sampled teachers (e.g., [30,23,28,25,18]), it limits our ability to draw generalizable conclusions. Instead, the strength of this mixed methods study lies in its ability to provide a nuanced understanding of teachers’ AWE implementation and perceptions within this specific context. This rich qualitative insight complements the quantitative data, offering a comprehensive view that can inform future research and experimental studies aimed at validating and expanding upon the factors we identified.

Study findings indicated that although teachers had generally positive perceptions of MI Write, teacher implementation was quite variable and minimal in some cases despite teachers’ voluntary participation in a randomized controlled study and having access to training and a detailed set of implementation expectations. Further, usage of some features—like peer review and the annotation tool—was near zero. In addition, although most teachers perceived MI Write as easy to us, there was greater variation in their perceptions of MI Write's usefulness and social desirability, particularly across school districts. Interview and focus group data helped to explain these quantitative results, pointing to broader contextual factors such as curricular alignment and the challenge of balancing MI Write implementation with curricular expectations as key influencing factors. In general, findings partially support the hypothesis of the Technology Acceptance Model [19,20], which posits that perceptions of a system's usability and usefulness are positively related to individuals’ intention and actual system use. Specifically, while negative perceptions of usability and usefulness—particularly usefulness—serve as a barrier to the use of AWE systems, it is noteworthy that positive perceptions alone did not guarantee high levels of utilization. This nuanced understanding highlights the complexity of AWE adoption, suggesting that other intervening factors not directly captured by the TAM's focus on system usability and usefulness play a critical role in translating positive attitudes into actual usage. The section that follows provides additional discussion of these points.

### 4.1 Ecological factors, not only system factors, influence AWE implementation

Results of this study highlight that teacher willingness, access to training, and generally positive perceptions of MI Write's usability and usefulness were not sufficient to produce consistently strong implementation, and none of the teachers met overall implementation expectations. Study findings are consistent with prior research that indicates that even when perceived positively, AWE tends to be utilized variably across districts, schools, and teachers, often leading to underutilization overall (e.g., [35,18,34]). Thus, study findings point to the importance of examining factors within the broader ecological context in which AWE implementation occurs.

First, curricular alignment appears to be a critical factor. Teachers appeared less likely to perceive an easy-to-use AWE tool as useful or socially desirable if it was not compatible with their curriculum or if using the tool conflicted with other instructional priorities. Prior research has identified curricular integration as an important factor influencing teachers’ AWE implementation and perceptions [30,27,33,34]. However, findings from this study go further in identifying specific curricular factors for stakeholders to attend to, including (a) the pacing of writing assignments across the school year; (b) the genre of writing assignments within the curriculum, specifically shorter response to text assignments or longer essay-length assignments; (c) teachers’ beliefs regarding, and their preferred mode of providing, feedback (see also [28,25]); (d) the accuracy and alignment of the automated scoring and feedback to teachers’ own feedback and grading scale; and (e) teacher and curricular priorities, specifically whether writing is a priority of its own or seen as furthering reading goals. These nuanced elements of curricular alignment appear to play a role in shaping the relationship between teachers’ use and perceptions of AWE systems like MI Write. In the present study, when such alignment was absent, teachers experienced an unwanted burden of helping students interpret scores and understand limitations of automated scoring, a burden reported by teachers in prior research as well [27,31,22,25]. Indeed, teachers were least likely to agree that MI Write reduced their grading workload.

Second, at the school and district level, the availability of support for AWE implementation is crucial. When teachers work with supportive leadership and have an organized peer support system, they demonstrate more positive attitudes towards AWE and higher implementation [32,33]. Several teachers in this study reported difficulties in implementing MI Write due to concurrent demands of implementing the StudySync curriculum. Teachers also expressed frustration with demanding and competing priorities, such as their curriculum prioritizing reading whereas the research study prioritized writing. Thus, findings suggest that implementation challenges are likely when multiple curricula and programs are layered, especially when teachers are not offered administrative support or guidance regarding priorities. To address these issues, implementers should consider ways to reduce demands on teachers (e.g., freeing teachers from committee work) or providing additional time and resources (e.g., an additional planning period) to facilitate teacher learning and effective AWE implementation.

Third, broader social factors, such as educational policies, must be considered. In this study, schools operated under various COVID-19 policies. Teachers reported implementation challenges stemming from teacher and student absences due to sickness or required quarantines, shifts from in-person to remote instruction, and the need to accelerate learning for students who experienced COVID-19-related learning loss. Although, these specific challenges may not be generalizable beyond the pandemic, they highlight the importance of examining how broader social and policy-level ecological factors can influence teachers' implementation and perceptions of AWE in future studies. Overall, more careful and direct consideration of these ecological factors in future research may lead to greater success implementing AWE.

### 4.2 Limitations and future directions

Several limitations should be considered when interpreting the results of the present study. First, the sample size was small, which is consistent with other mixed-methods studies of AWE, but this limits the generalizability of the findings. Future research using larger samples of middle school ELA teachers in other school districts should be conducted to replicate the themes and factors identified in the current study. Such research with larger samples should also consider additional quantitative analyses (e.g., regression) to further examine the relationship between teachers’ social validity perceptions and fidelity of implementation (e.g., Wilson, Zhang et al.'s [46] study of students’ AWE perceptions). Second, the interview protocol did not include questions that explicitly asked teachers to discuss their perceptions of MI Write's social desirability. Although the protocol indirectly captured teachers’ perceptions of social desirability through examining contextual features that facilitated and prevented teachers’ MI Write usage, explicit questions about social desirability could have facilitated further understanding the variability in survey responses around this construct. Also, it is important to acknowledge that this study was conducted over the course of one academic year only. Although long-term AWE implementation is an important avenue of research, it is likely that teachers who are long-term adopters of AWE hold positive perceptions of AWE or else they would have stopped using the software. Thus, long-term users likely represent a biased sample of the teacher population. Hence, our decision to investigate relationships between implementation and perceptions with teachers in their first year of AWE adoption represents a strength of our study, allowing us to explore relationships in a more diverse teacher sample. Nevertheless, it must be acknowledged that this study did not explore changes in teachers’ perceptions over time. Lastly, the current study exclusively examines teachers’ use and perceptions of AWE, omitting student perspectives. A significant avenue for future research could be to investigate the reciprocal influence between teacher and student perceptions of AWE.

## 5 Conclusion

This study examined the relatively unexplored relationship between teacher implementation and perceptions of AWE. Its focus on middle school teachers also is a strength; most AWE research focuses on higher education. Other strengths of this study include being situated within a rigorously conducted randomized controlled trial, utilizing mixed methods, and utilizing a sample of teachers from several school districts and all new to AWE. Despite the small sample size, practically, our findings underscore the necessity for nuanced AWE integration strategies that attend to a broader ecology of factors including, but not limited to, system-level factors. By identifying such ecological factors that appeared to influence teachers’ implementation and perceptions of MI Write, this study offers insights that can guide future efforts to enhance the effectiveness and adoption of AWE systems.

The study results indicate that curricular alignment is a key factor in the successful implementation of AWE systems. Curriculum publishers may have an advantage because they can develop AWE tools that align directly with their own ELA curricula, offering a package solution to school districts. For developers of stand-alone AWE systems—those created independently of a particular curriculum like MI Write—enhancing customization options may have beneficial effects on implementation and outcomes. Such customization might include not only adapting the system to different types of writing assignments but also allowing flexibility in the traits evaluated and feedback provided.

However, this raises a fundamental design dilemma: should AWE systems prioritize adherence to best practices in scoring, feedback, and writing assessment, or should they be maximally customizable to fit existing instructional practices? The first approach promotes accurate scoring, effective feedback, and supports best instructional practices, such as peer review, but may not align with current teaching methods. On the other hand, a highly customizable system would better integrate with diverse classroom practices but could limit the system's potential to significantly improve the frequency and quality of writing instruction, which is often lacking (see [51]). Although designing systems that integrate seamlessly into existing instructional contexts is essential, it is equally important to leverage AWE's potential to elevate writing instruction and support evidence-based practices.

Regardless of whether the adopted AWE system is curriculum-specific, stand-alone and uncustomizable, or stand-alone and customizable, ELA curriculum leaders play a critical role. They must establish and enforce clear expectations for teachers’ use of AWE, while also ensuring that teachers are well-trained, supported, and provided with the time and resources needed to implement the system effectively.

## Conflict of interest statement

The following authors declare no conflicts of interest relevant to this work: Joshua Wilson, Amanda Delgado, Tania Cruz Cordero, Matthew C. Myers, Andrew Potter, Saimou Zhang. The following authors are or were employed by Measurement Incorporated at the time of the study: Corey Palermo, Halley Eacker, Jessica Coles.

This work was supported, in whole or in part, by the Bill & Melinda Gates Foundation [INV-006,167]. Under the grant conditions of the Foundation, a Creative Commons Attribution 4.0 Generic License has already been assigned to the Author Accepted Manuscript version that might arise from this submission. The opinions expressed in this paper are those of the authors and do not represent the views of the Foundation, and no official endorsement by this agency should be inferred.

The data that support the findings of this study are available from the corresponding author upon reasonable request.

During the preparation of this work the authors used ChatGPT to revise and edit portions of the text to improve readability. After using this tool/service, the authors reviewed and further edited the content as needed and take full responsibility for the content of the publication.

^a^Jessica Coles is now at WestEd.

^b^Halley Eacker is now at Lansingburgh Central School District.

## CRediT authorship contribution statement

**Joshua Wilson:** Writing – review & editing, Writing – original draft, Supervision, Project administration, Methodology, Investigation, Funding acquisition, Formal analysis, Conceptualization. **Amanda Delgado:** Writing – review & editing, Writing – original draft, Methodology, Investigation, Formal analysis, Conceptualization. **Corey Palermo:** Writing – review & editing, Supervision, Resources, Project administration, Methodology, Funding acquisition, Conceptualization. **Tania M. Cruz Cordero:** Methodology, Investigation. **Matthew C. Myers:** Investigation. **Halley Eacker:** Supervision, Resources, Project administration, Investigation. **Andrew Potter:** Investigation. **Jessica Coles:** Resources, Investigation. **Saimou Zhang:** Writing – original draft, Conceptualization.

## Declaration of competing interest

The following authors declare no conflicts of interest relevant to this work: Joshua Wilson, Amanda Delgado, Tania Cruz Cordero, Matthew C. Myers, Andrew Potter, Saimou Zhang. The following authors are employed by Measurement Incorporated: Corey Palermo, Halley Eacker, Jessica Coles.

## Appendix Supplementary materials

Image, application 1
