# Supplementary material for: Middle school teachers’ implementation and perceptions of automated writing evaluation
Source: Comput Educ Open. 2024 Dec;7:None. doi: 10.1016/j.caeo.2024.100231 (PMC11656462; doi:10.1016/j.caeo.2024.100231)
Supplement: Supplementary file 1 [file mmc1.docx]

# **Appendix A – Interview/Focus Group Protocol**

## Usability

**Usability for Teachers**

1. Do you think MI Write is easy for teachers to use? Why or why not?
   1. Prompts:
      1. Providing teacher feedback comments (and reading student writing)
      2. Monitor student and class progress
2. If you experienced challenges using MI Write, how did you attempt to address those challenges? Do you have any suggestions for improving the usability of MI Write for teachers?

**Usability for Students**

1. Do you think MI Write is easy for students to use? Why or why not?
   1. Prompts:
      1. Revise essays using feedback
      2. Conduct peer review
2. Were there groups of students who particularly struggled to use MI Write? If so, which groups of students struggled, and why do you think they struggled?
   1. Prompts:
      1. struggling readers or writers (ability)
      2. students with language differences (language status)
      3. students with learning disabilities (disability status)
3. If your students experienced challenges using MI Write, in what ways did you attempt to make MI Write easier for your students to use?

## Usefulness

**Teacher Usefulness**

1. We’re interested in learning how MI Write may have helped you as a teacher. In what ways, if any, was MI Write useful for you as a teacher?
   1. Prompts:
      1. Does it support conferencing?
      2. Does it result in you providing different amounts of types of feedback?
      3. Does it help you monitor the progress of your class or students?

*If they say MI Write was not useful*: Can you explain why MI Write was not useful to you

as a teacher?

1. Did using MI Write help improve your confidence in teaching writing? Why or why not?
2. What did you find useful about the monthly professional development/coaching sessions? What didn’t you find useful about those sessions?
3. Do you have any suggestions for improving the usefulness of the monthly professional development/coaching sessions?

**Student Usefulness**

1. We’re interested in how MI Write may have helped your students. In what ways, if any, was MI Write useful to your students?

*Prompt teachers to elaborate and provide examples if they don’t provide them.*

- 1. Prompts:
     1. Does it improve their confidence?
     2. Did it improve the quality of student writing?
     3. Did you observe any undesirable changes?

1. Do you think MI Write is equally useful for students of different ability, language, or racial/cultural backgrounds?

## Facilitators and Barriers

1. What factors facilitated your ability to implement MI Write in your classroom?

*Allow teachers to answer freely and then display Slide #1 (see below). Ask,* “In addition to what you just said, were any of these factors facilitators for you this year? If so, please discuss 1 or 2 additional factors from this list that were particularly helpful. When you respond, please say the corresponding number of the factor you discuss.”

1. What were the barriers impacting your ability to implement MI Write in your classroom?

*Allow teachers to answer freely and then display Slide #2 (see below). Ask,* “In addition to what you just said, were any of these factors barriers for you this year? If so, please discuss 1 or 2 additional factors from this list that posed a significant barrier. When you respond, please say the corresponding number of the factor you discuss.”

1. To what extent were the barriers you just discussed related to COVID’s impacts on education (COVID, COVID climb) versus barriers that would occur even in a “normal” school year?

## Utilization and Adherence to Program Model

1. Did you change your approach to writing instruction as a result of using MI Write? Were these changes positive or negative?
2. Did you have your students use MI Write’s planning tools (for example, graphic organizers)? If so, how did these tools fit with the instruction on planning that you provided?
3. How did you have your students approach revising through MI Write?
4. This year we provided teachers with monthly expectations for using MI Write with your students. Were these expectations feasible? If not, how much exposure to MI Write would be feasible to manage but also benefit students?

# Slide 1

**Potential Facilitators**

1. Access to timely technical assistance and support
2. Support from building and district leadership
3. Curriculum materials that emphasize similar types of writing as evaluated by MI Write
4. Fluency/Skills with technology
5. Resources made available through the MI Write Help Center or my coach
6. Sufficient time for writing instruction
7. Interest or motivation in learning about automated essay evaluation and feedback
8. Support from special education co-teachers in my classroom
9. Learning from teachers in my building who are also implementing MI Write

# Slide 2

**Potential Barriers**

1. Time for writing instruction in my daily schedule
2. Students’ reading skills
3. Need for additional professional development or coaching opportunities
4. Technical challenges of using MI Write
5. Lack of alignment with ELA/Writing curriculum and MI Write
6. Student absences and teacher absences
7. Student behavior/classroom management
8. Multiple, layered and/or competing priorities at the district or school levels
9. Lack of prep time/space for meetings
